# Improved Thin-Kerf Processing in C_f_/SiC Composite by Waterjet-Guided Nanosecond Laser Decreases Oxidation and Thermal Effect

**DOI:** 10.3390/ma18071560

**Published:** 2025-03-29

**Authors:** Jiayu Wang, Guangyi Zhang, Qiaoli Wang, Youmin Rong, Chaochao Zhao, Chunguang Chen, Binying Bao, Wenwu Zhang, Liyuan Sheng

**Affiliations:** 1 Key Laboratory of Aero Engine Extreme Manufacturing Technology of Zhejiang Province, Ningbo Institute of Materials Technology and Engineering, Chinese Academy of Sciences, Ningbo 315201, China; wangjiayu@nimte.ac.cn (J.W.); chenchunguang@nimte.ac.cn (C.C.); baobinying@nimte.ac.cn (B.B.); zhangwenwu@nimte.ac.cn (W.Z.); 2 Faculty of Mechanical Engineering and Mechanics, Ningbo University, Ningbo 315211, China; 3 University of Chinese Academy of Sciences, Beijing 100049, China; 4 PKU-HKUST ShenZhen-HongKong Institution, Shenzhen 518057, China; wangqiaoli@163.com (Q.W.); chaoaooo@163.com (C.Z.); 5 School of Mechanical Science and Engineering, Huazhong University of Science and Technology, Wuhan 430074, China; 6 Shenzhen Institute, Peking University, Shenzhen 518057, China

**Keywords:** waterjet-guided laser, C_f_/SiC composite, surface morphology, chemical composition, heat-affected zone

## Abstract

As a hard and brittle material, the processing of C_f_/SiC ceramic matrix composites (CMCs) faces significant challenges, especially in the processing of small-sized shapes. To address this challenge, laser processing with gas-assisted nanosecond laser (GNL) and waterjet-guided nanosecond laser (WNL) modes were applied to fabricate thin kerfs in the C_f_/SiC composite. The surface morphology, microstructure, and chemical composition of the processed C_f_/SiC composite were investigated comparatively. The results revealed that the coupling of helium in the GNL mode laser processing could make full use of the laser energy, but resulted in spattering in the kerf margin and a recast layer in the kerf surface, accompanied by obvious oxidation, while the coupling of the waterjet in the WNL mode laser processing decreased the oxidation significantly and removed the remelting debris, which produced a clear and flat kerf surface. Due to the taper caused by laser energy dissipation, the single-path laser processing in the C_f_/SiC composite had a limited depth. The maximum depth of the kerf prepared by single-path laser processing with the GNL mode was about 328 μm, while that with the WNL mode was about 302 μm. The multi-path laser processing with the GNL and WNL modes could fabricate a through kerf in the C_f_/SiC composite, but the coupling medium obviously influenced the surface morphology and microstructure of the underlying region. The kerf surface prepared by the GNL mode had a varied surface morphology, which transited from the top layer, covered with oxide particles and some cracks, to the bottom layer, featured with micro-grooves and small oxides. The kerf surface prepared by the WNL mode had a consistently smooth and clean morphology featured with broken carbon fiber and residual SiC matrix. The slow laser energy dissipation and open environment in the GNL mode resulted in a bigger HAZ and relatively serious oxidation, which caused local phase transformation and microstructure degradation. The isolation condition and rapid cooling in the WNL mode decreased the HAZ and restrained the oxidation, almost keeping the original microstructure. The thicknesses of the HAZ in the GNL- and WNL-processed C_f_/SiC composite were about 200 μm and 100 μm, respectively. The WNL-processed C_f_/SiC composite had a lower oxidation and thermal damage surface, which is instructive for the processing of the C_f_/SiC composite.

## 1. Introduction

Recently, the worsening environmental pollution and energy crisis have accelerated the development and application of innovative materials and its corresponding technology [1,2]. Particularly in the fields of aerospace and transportation, the requirements for improving the energy utilization ratio and conserving energy demand lightweight designs and materials [3,4]. Ceramic matrix composite (CMC) is a kind of innovative material, having a high specific strength, high melting point, excellent corrosion resistance, and high chemical stability, which can be widely used in aerospace, transportation, and other fields [5,6]. Among the CMCs, the C_f_/SiC composite is the most representative one because of its combination of high-strength carbon fiber and the excellent heat-resistant SiC matrix [7,8]. Thus, the C_f_/SiC composite demonstrates attractive properties, such as a low density, high oxidation resistance, excellent high-temperature strength, and excellent abrasion resistance, showing great potential application in many important industrial fields [9,10]. In aerospace, it is used in engine combustion chambers [11], nozzles [12], and turbine blades and blade disks [13,14]. In transportation, it is applied in the braking components of aircrafts, high-speed trains, and high-end automobiles [15,16]. Additionally, it is widely used in the field of opto-mechanics, particularly in mirror substrates and precision structural components [17]. However, to achieve these applications, high-precision and high-quality processing of the C_f_/SiC composite is the fundamental premise, which determines the final performance. As a kind of hard and brittle material, it is difficult to obtain the well-machined C_f_/SiC composite component by traditional processing techniques. Furthermore, the anisotropy and heterogeneity features induced by the fiber structure also exert an obvious influence on the processed surface of the C_f_/SiC composite [18,19].

In general, the traditional processing methods for C_f_/SiC composites mainly include machinery processing, abrasive waterjet machining, and electrical discharge machining (EDM) [20]. Machine processing has the advantage of a low cost and mature equipment, but the integrity of processed surfaces is difficult to control. Due to the high stress induced by cutting forces, defects such as cracks, fiber breakage, and pullout often appear in processed surfaces [21]. Abrasive waterjet machining can achieve a relatively better processing surface, and its efficiency is also acceptable, but the high impact force caused by the high-speed waterjet always causes fiber pullout and chipping, especially in the processing of thicker composites [22]. Comparatively, the EDM does not have the problem caused by high impact stress, and the processed composite surface has a high quality, but the electrical discharge feature in its micro-region requires the excellent conductivity of the processed composite. Though carbon fiber can improve the electrical conductivity of the SiC matrix, it is impossible to ensure the production of the micro-region, and the electrical insulation feature of the SiC is responsible for the great difficulty in the application of the EDM in its processing [23].

As a kind of non-traditional processing technique, the laser demonstrates tremendous advantage in materials processing due to its high energy density, ultrafine processing area, high precision, non-contact processing, etc. Therefore, it is widely utilized in the processing of superalloys, ceramics, composites, refractory metals, and other difficult-to-process materials. Nowadays, there are many studies on the laser processing of C_f_/SiC composites [24,25,26]. Wang et al. [24] performed laser ablation experiments on a C_f_/SiC composite using a continuous-wave (CW) laser, which revealed the presence of an ablation threshold of about 288 MW/m^2^ for this material. Specifically, the carbon fiber and SiC matrix also had greatly differing laser ablation thresholds, and thus microscopic ablation morphologies, such as the “shoot tip” and “matrix pore”, were formed on the ablated C_f_/SiC composite surface. Yan et al. [25] investigated the influence of water and air media on the nanosecond laser ablation behavior of a C_f_/SiC composite, which demonstrated the presence of an obvious heat-affected zone on the sides of the processed groove in the different states. Even though the water medium significantly suppressed the thermal effect, recasting was also observed. With the increase in the laser power and processing numbers, the dimension of the heat-affected zone tended to increase in all medium environments. Zhang et al. [27] processed micropores in C_f_/SiC composites using a CW laser and observed a large amount of residue debris in the sidewalls of the micropores, indicating extensive oxidation during laser ablation. Due to the relatively low quality, the laser-processed micropores cannot be applied directly. Therefore, to apply the laser-processed C_f_/SiC composites directly, it is critical to solve the problem of the redundant thermal effect and oxidation, which determine the quality of the processed components.

According to previous studies [28,29], it can be found that water is the most effective medium to remove redundant heat during the processing of materials. If the water is coupled with a laser, it becomes an optimal choice for the processing of the C_f_/SiC composite. As a result, waterjet-assisted laser processing was developed, which coupled the laser beam into the micro-sized water beam and transmitted it to the material surface by reflection [30,31]. Due to the scouring and cooling effects of the waterjet, its assisted laser processing could minimize the thermal damage and obtain a cleaner surface [32,33]. Chao et al. [34] comparatively studied the processing of grooves with a high depth-to-width ratio in the Ti-6Al-4V alloy by waterjet-guided and dry lasers, demonstrating a neater kerf and smaller heat-affected zone obtained by the waterjet-guided laser. Moreover, the waterjet-guided laser processing could increase the average microhardness of the processed surface by 55%, which was obviously higher than that obtained by the dry laser processing. Sun et al. [35] performed the waterjet-guided laser cutting of a CFRP composite and revealed the mechanism of the heat-affected zone variation induced by water assistance. Comparatively, the waterjet-guided laser processing could take full use of the high energy density of the laser and thermal dissipation of water, thereby realizing highly qualified and precise processing. It is anticipated that this kind of processing could be well suitable to the C_f_/SiC composite, with high quality requirements.

To meet the surface quality requirements of the materials used in applications like blades and microfluidic channels, a waterjet-guided nanosecond laser (WNL) was employed to create a thin kerf in the C_f_/SiC composite, aiming to achieve a high-quality processed surface. For comparative study, a gas-assisted nanosecond laser (GNL) was employed to process the same C_f_/SiC composite. The surface morphology, microstructure, and chemical composition of the processed C_f_/SiC composite were characterized to explore the material removal mechanism and its influencing effect. This study is expected to provide a feasible laser processing technique for the high-quality machining of C_f_/SiC composites.

## 2. Materials and Methods

### 2.1. Material

**Figure 1:**
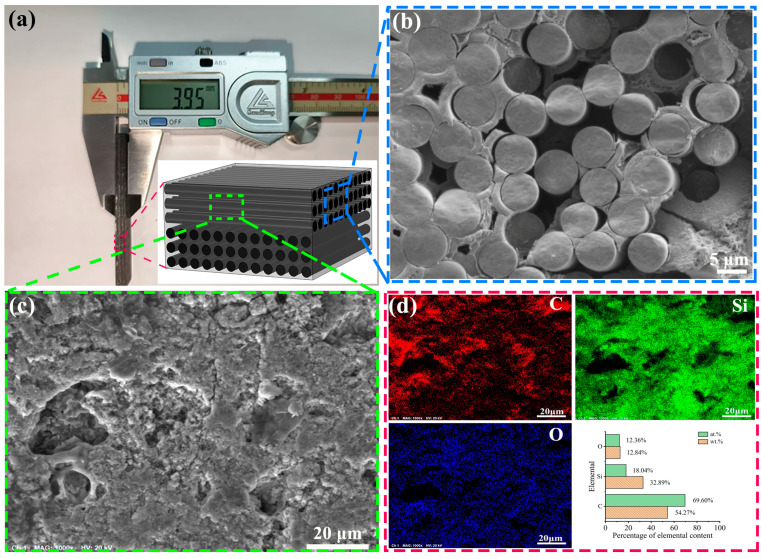
Macrograph, structure, and composition of the chemical distribution of the used C_f_/SiC specimen: (**a**) Macrograph of the C_f_/SiC specimen (inset image shows a schematic diagram of the carbon fiber arrangement); (**b**) Microstructure of the carbon fiber bunch; (**c**) Microstructure of the composite with carbon fibers with an axial direction perpendicular to the observation area; (**d**) EDS analyses on the elements and their distributions in (**c**).

The C_f_/SiC composite plate used in the present research had a 3D needle-punched structure and was produced by Zhejiang Hangyin New Material Technology Company. The thickness of the C_f_/SiC composite plate was about 4 mm, and was cut into small specimens with a size of 60 mm × 10 mm, as shown in Figure 1a. The carbon fibers were regularly arranged in an intersected direction, and the layer thickness was about 300~400 μm. The typical SEM observation on the carbon fiber bunch showed the relative regular distribution of the carbon fibers with a homogeneous interval distance, as shown in Figure 1b. The typical SEM observation on the composite with carbon fibers with an axial direction perpendicular to the observation area exhibited a non-compact microstructure containing some micropores with differed sizes, as shown in Figure 1c. The EDS analyses on the cross-section of the composite with carbon fibers with an axial direction indicated an inhomogeneous elemental distribution, as shown in Figure 1d. Such a phenomenon should be partly attributed to the presence of porosity, while the carbon fiber and SiC also caused the different elemental distribution. That is why there was a high proportion of carbon. The presence of oxygen should be ascribed to its introduction during the fabrication of the C_f_/SiC composite [36]. The detailed physical properties of the C_f_/SiC composite are shown in Table 1. Due to the initial fabrication of the carbon fiber, there was a pyrolytic carbon (PyC) interfacial layer on its surface, with thickness of about 300 nm. The carbon fibers had diameter of 6~8 μm, and their volume fraction was about 40%. The porosity of the C_f_/SiC composite was about 18%, which resulted in its lower density than the theoretical value. The C_f_/SiC composite specimens were ultrasonically cleaned in ethyl alcohol before the laser processing.

### 2.2. Laser Processing

**Figure 2:**
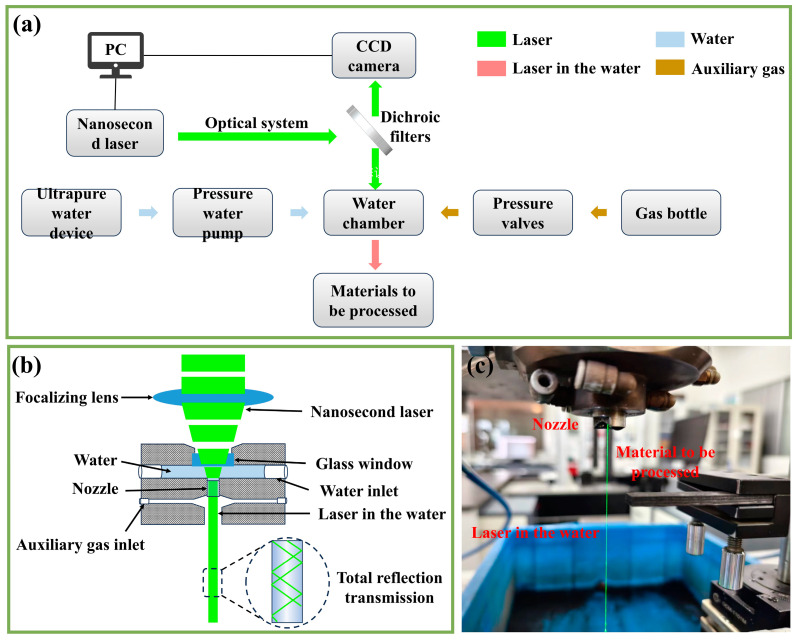
Schematic diagram of the laser processing system and corresponding equipment: (**a**) The framework of the laser processing with the assistance of water and gas; (**b**) The structure of the waterjet-guided system; (**c**) The equipment for the WNL processing.

To realize the laser processing with different media, a self-developed laser processing platform was applied in the present research. As shown in Figure 2a, the laser processing platform was mainly composed of a three-axis motion system, controlling system, nanosecond laser, scanning mirrors, charge-coupled device (CCD) camera, and optical transmission system. The solid-state Nd/YAG laser (Solide Laser Co., PR-532-5-B, Suzhou, China) was used to generate a nanosecond pulsed laser with a wavelength of 532 nm, a pulse width of 75–90 ns, a pulse frequency of 30 kHz, and an average power of 20 W. To achieve the coupling of the waterjet and the laser beam, a waterjet-guided system was designed, as shown in Figure 2b. It was comprised of a water inlet, gas inlet, and waterjet path, by which the water with a certain pressure could form a linear and fine water bundle. The waterjet-guided system was fixed with the laser processing platform, as shown in Figure 2c. During the WNL processing, the laser beam was transmitted from the glass window of the waterjet-guided system to the waterjet, and was then reflected in it to the specific processed region. In order to minimize the loss of the laser by impurity scattering, ultrapure water was used, which was sent by a water pump with a specific pressure to generate a stable waterjet. Additionally, the helium entered through the gas inlet with a certain pressure, whose stream helped to increase the laminar flow length and prevent sputtering phenomena from disturbing the waterjet. When the processing was changed to the GNL mode, the water valve was closed and the helium blew away the residual water, forming the weak helium stream along the laser direction.

**Figure 3:**
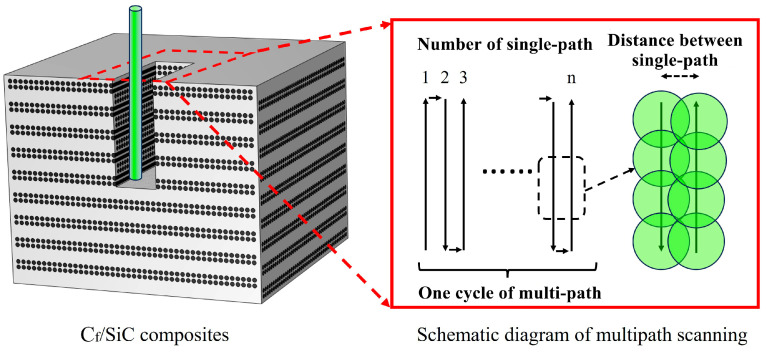
Schematic diagram of laser processing the kerf in the C_f/_SiC composite: (**a**) The processed kerf in the C_f/_SiC composite; (**b**) The multi-path reciprocating route for the laser scanning.

The aim of the present research was to process a thin kerf in the C_f_/SiC composite with a dimension of 100 µm × 10 µm, as shown in Figure 3a. To achieve this objective, the C_f_/SiC composite was processed on the self-developed laser processing platform using the WNL and GNL modes. Except for the coupling medium, the two kinds processing modes adopted the same laser parameters and laser scanning routes. The multi-path reciprocating route composed of 9 single-path reciprocating conditions was chosen for the laser scanning, which had about a 30% laser spot overlap, as shown in Figure 3b. Such a multi-path reciprocating route was repeated to obtain the thin kerf in the C_f_/SiC composite. During the laser processing, the laser parameters described above were used, and the other parameters were set as follows: a waterjet velocity of 110 m/s, a helium pressure of 0.2 MPa, a nozzle diameter of 100 µm, a scanning speed of 3 mm/s, a laser spot diameter of 30 µm, and a distance between the parallel paths of 70 µm, as shown in Table 2. The kerfs were processed in the C_f_/SiC composite by the WNL and GNL modes with the same parameters. To verify the effect of the single-path laser processing, the C_f_/SiC composite was processed by the WNL and GNL modes with different numbers of single-path routes. The obtained specimens were ultrasonically cleaned in ethyl alcohol to clear away the impurities and debris.

### 2.3. Characterization

The specimens used for characterization were cut from the laser-processed C_f_/SiC composite. To observe the surface morphology of the kerf and the corresponding heat-affected zone (HAZ), the C_f_/SiC composites processed by different laser modes were cut along the center-line and cross-sectional positions of the kerfs. The obtained specimens were ultrasonically cleaned in ethyl alcohol for 10 min to remove the impurities. Afterwards, all of the specimens were dried at room temperature. The size and shape of the kerfs were analyzed by a laser scanning confocal microscope (CLSM, Keyence VX-200, Keyence Co., Osaka, Japan). The surface morphologies and elemental distributions of the laser-processed composites produced by different modes were observed and analyzed using scanning electron microscopy (SEM, FEI Quanta FEG 250, Hillsboro, OR, USA) coupled with an energy-dispersive spectrometer (EDS, Oxford X-MAX 50, Oxford, UK). To examine the detailed variations in the surface chemical bonds, X-ray photoelectron spectroscopy (XPS) was employed to analyze the laser-processed composite surface.

## 3. Results and Discussion

### 3.1. Morphology and Depth of Kerf by Single-Path Laser Processing

**Figure 4:**
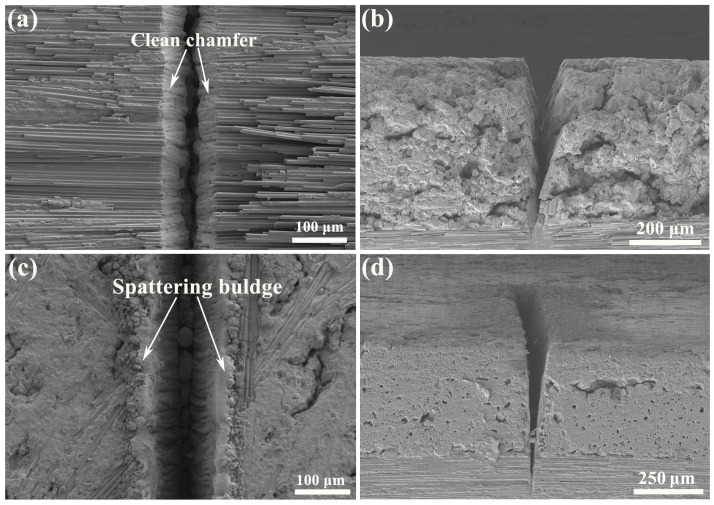
Typical morphology of the kerf by laser processing with different modes: (**a**) Surface morphology of the kerf processed by WNL; (**b**) Cross-sectional morphology of the kerf processed by WNL; (**c**) Surface morphology of the kerf processed by GNL; (**d**) Cross-sectional morphology of the kerf processed by GNL.

The typical morphology of the kerf prepared by single-path laser processing was observed by SEM and the results are given in Figure 4. Clearly, the differed coupling medium influenced the processed kerf. As shown in Figure 4a, the kerf processed by WNL demonstrates a clean surface, but there is an obvious chamfer on the top. Such a morphology should be ascribed to the extremely high cooling effect caused by the coupling of the laser and water. Due to the high energy density of the laser, its irradiation on the C_f_/SiC composite sharply enhances the temperature of the local region, while its dwell time is short. Thus, the waterjet cools down the irradiated region rapidly, which produces a great cooling rate and the cracking of the micro-region. Moreover, the kerf margin is not linear and exhibits a zigzag-like shape. The cross-sectional morphology of the kerf indicates the high taper angle generated during the WNL processing, as shown in Figure 4b. Such a phenomenon implies that the single-path laser processing could not realize the preparation of a kerf with a high depth because of the dissipation of the laser energy. Especially in the position exceeding 85% of the whole depth, the width of the kerf becomes very small. The morphology of the kerf processed by GNL demonstrates a relatively different state, as shown in Figure 4c. There is an obvious bulge along the kerf margin, which should be the outlet of the remelted composite. Its surface exhibits a typical slope with winkled features, indicating the formation of a recast layer. At the bottom of the kerf, solidified molten drops are found as well, which also confirms the difficulty of expelling the debris. The observation of the cross-section of the kerf reveals that its depth is a little higher that of the WNL-processed kerf, as shown in Figure 4d. Moreover, its kerf surface is relatively straight. Such a result also verifies the laser energy dissipation by the waterjet.

**Figure 5:**
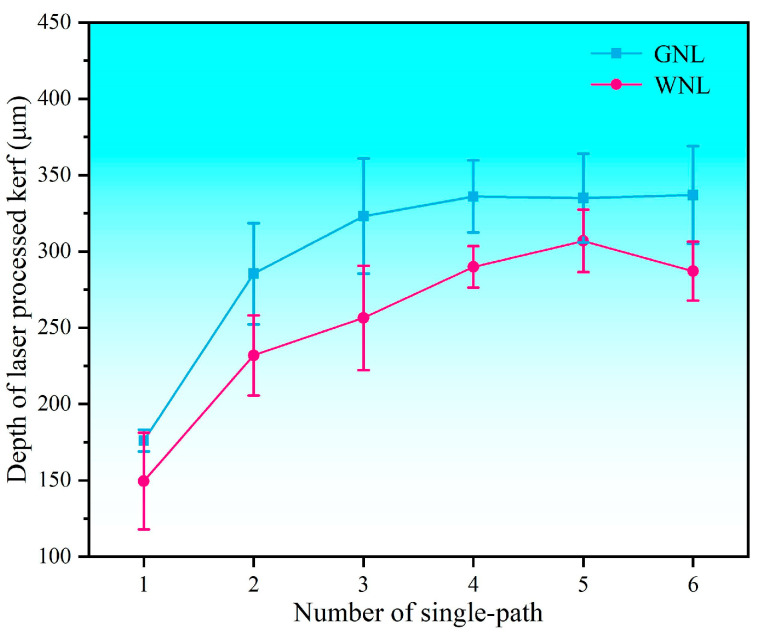
Variation in the kerf depth with the number of single-path laser processing routes in the WNL and GNL modes.

Based on the observation of the kerf prepared by single-path laser processing, it can be found that the taper angle caused by laser energy dissipation results in the limited depth of the kerf in the C_f/_SiC composite. As with the designed experiment above, there could be several single-path routes in one cycle of multi-path reciprocation. As a result, it is necessary to determine the optimal number of single-path laser processing routes in one cycle of multi-path reciprocation. The statistical analyses on the depth of laser-processed kerfs in the C_f/_SiC composite by WNL and GNL are shown in Figure 5. Clearly, the GNL obtains a deeper kerf than the GNL in the C_f/_SiC composite. The kerf depth prepared by GNL with one single-path laser processing is about 176 μm. With the increase in the single-path number, the kerf depth is increased, and the values of two single-path, three single-path, and four single-path laser processing routes are 285 μm, 323 μm, and 328 μm, respectively. Afterwards, the kerf depth in the GNL-processed C_f/_SiC composite fluctuates a little in scope, which indicates that the further increase in single-path laser processing would obtain no effect. The kerf depth prepared by WNL with one single-path laser processing route is about 150 μm. The increased number of single-path laser processing routes enhance the kerf depth gradually, and the kerf depths of two single-path, three single-path, four single-path, and five single-path laser processing routes are 232 μm, 256 μm, 281 μm, and 302 μm, respectively. Interestingly, the depth of six single-path laser-processed kerfs is 261 μm, which is lower than that with fewer laser processing numbers. These results imply that the kerf depth is not linearly related with the single-path number of laser processing routes. During the fourth to sixth processing routes, the depth of the kerf did not increase significantly, and the kerf processing encountered a depth bottleneck.

Specifically, the laser source used in the present research is a Gaussian beam, even though it was transmitted in a waterjet, which results in an uneven energy distribution in the laser spot. Such a feature causes the taper morphology of the laser-processed shape, which is also the main reason limiting the processing depth [37,38]. During the laser processing of the C_f_/SiC composite by the GNL mode, the initially formed taper increases the laser-irradiated area but further decreases the energy density, which lowers the laser processing efficiency. The same problem also exists in the laser processing of the C_f/_SiC composite by the WNL mode. Moreover, the narrower bottom of the kerf destroys the waterjet shape, which weakens the coupling effect of the laser and the waterjet [36,39]. Therefore, it is difficult for the laser processing to achieve the kerf with a certain depth by the single-path method. In the present research, a blind kerf or a through kerf with a small width is prepared by multi-path laser processing, which makes full use of the laser energy, thus obtaining a regular shape.

### 3.2. Morphology and Microstructure of Kerf by Multi-Path Laser Processing

**Figure 6:**
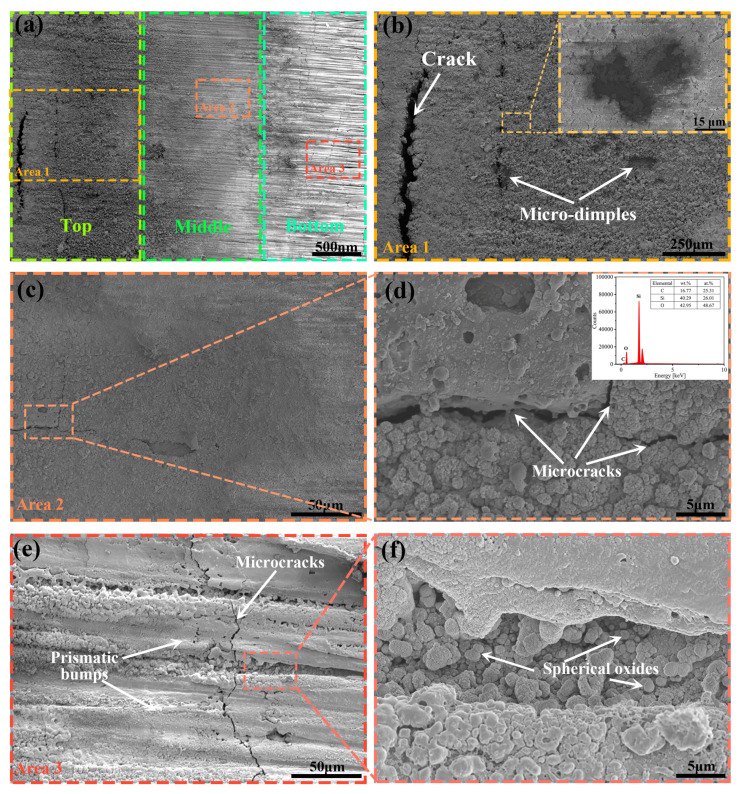
SEM observations on the kerf surface prepared by laser processing with the GNL mode in the C_f_/SiC composite: (**a**) A brief view of the kerf surface; (**b**) Morphology of the top region of the kerf surface (the inset image shows micro-dimples with the remelted feature); (**c**) Morphology of the middle region of the kerf surface with micro-cracks and local exfoliation; (**d**) Formation of small oxides with a cauliflower-like morphology (the inset image shows the EDS result of this region); (**e**) Morphology of the bottom region of the kerf surface with a micro-groove morphology; (**f**) Formation of small oxides at the bottom of the micro-grooves.

Based on the single-path laser processing of the C_f/_SiC composite, the fabrication of the through kerf in this kind of composite should adapt multi-path laser processing, which decreases the taper shape by progressive processing. Laser processing with the WNL and GNL modes was applied to prepare the thin kerfs in the C_f/_SiC composite with a total thickness of 4 mm. The morphology of the kerf surface prepared by laser processing with the GNL mode is shown in Figure 6. A brief observation reveals that there are three different features in the kerf surface, which can be defined as the top, middle, and bottom regions, as shown in Figure 6a. In the top region, a sand-like morphology is its main feature, with some cracks. In the middle and bottom regions, the surface morphology transits from sand-like to micro-groove features. A detailed observation of the top region exhibits that it is covered with a large number of spherical particles, as shown in Figure 6b. These spherical particles demonstrate the diversified dimension with a scope of several to scores of micrometers. Moreover, a crack with a large size is also observed, which indicates the high inner stress generated in the surface layer. Micro-cracks and micro-dimples can also be observed, implying the differed laser ablation behavior in local areas. According to previous studies [39,40,41], the rapid cooling of small melt drops produce spherical particles due to the small surface energy. The inset image shows the morphology of the micro-dimples, which indicates the molten pool feature. It can be deduced that the relatively larger melt drops aggregated there result in the remelting of the adjacent matrix, thus producing the micro-dimples.

The observation on the middle region exhibits ultrafine particles and their small-groove features, as shown in Figure 6c. Clearly, some micro-cracks and local exfoliation can be observed as well, which indicates the interface stress formed under the surface layer. Further observation of the region with micro-cracks reveals that they mainly extend in the surface layer and separate in the oxidizing layer, which releases part of the inner stress, as shown in Figure 6d. The inset EDS analysis of this region confirms that oxides are the main components. The decreased size of the oxide particles indicates that the influence of oxygen is weakened because of the limited exchange of air. That is also the crucial reason for the kerf surface transiting from oxide particles to a micro-groove morphology.

When the observation region changes to the bottom region, its morphology changes obviously, as shown in Figure 6e. The micro-grooves become the main feature, and there are some small particles formed in the ridge of the bottom region. In addition, micro-cracks vertical to the micro-grooves can be observed, implying the great inner stress in the surface layer. Further observation of the bottom region or the micro-grooves reveals the formation of oxides with a mushroom-like morphology and different sizes, as shown in Figure 6f. Such a morphology indicates that the weakened oxygen influence could amplify the Gaussian distribution feature of the laser energy in the processed kerf surface. When the oxygen exchange is sufficient, the laser processing of the C_f/_SiC composite mainly comprises vaporization, remelting, and oxidation. The center of the laser-irradiated region vaporizes, and the adjacent region remelts and oxidizes, which leads to an oxide surface with a sand-like morphology. As the laser processing proceeds, oxygen becomes insufficient, which weakens the oxidation and results in the rapid solidification of the remelted composite, forming the micro-groove morphology. The weak air exchange also produces small oxides in the local regions.

**Figure 7:**
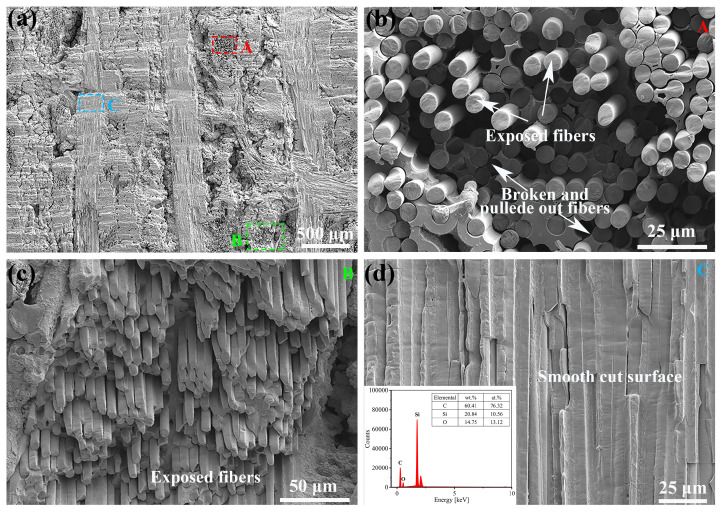
SEM observation of the kerf surface prepared by laser processing with the WNL mode in the C_f_/SiC composite: (**a**) A brief view of the kerf surface; (**b**) Morphology of the carbon fibers with an axial direction vertical to the laser beam; (**c**) Morphology of the carbon fibers having a certain angle with respect to the laser beam; (**d**) Morphology of the carbon fibers parallel to the laser beam (the inset image shows the EDS analysis of this region).

Different from the GNL-processed C_f_/SiC composite, the presence of the waterjet well eliminates the effect of the oxygen and the thermal effect. As shown in the Figure 7a, the kerf surface in the laser-processed C_f_/SiC composite with the WNL mode exhibits a relatively clean and homogeneous feature without a distinct difference in morphology. However, the arrangement direction of the carbon fibers exerts some influence on the kerf surface. The region with carbon fibers with an axial direction parallel to the laser beam exhibits a relatively smooth and flat surface, while the region with carbon fibers with an axial direction vertical to the laser beam exhibits a relatively rough surface with some randomly distributed exfoliation and pits. Further SEM observations of the region with carbon fibers with an axial direction vertical to the laser beam reveal the diversified carbon fiber morphology, as shown in Figure 7b. The carbon fibers demonstrate an inconsistent fractured surface, which includes breaking inside of the SiC matrix, breaking outside of the SiC matrix, and breaking with the SiC matrix. Such a morphology should be ascribed to the anisotropy of the carbon fiber in heat transfer, and the differing heat transfer behavior between the carbon and the SiC matrix [42]. This leads to the different temperature distribution in the carbon fiber and the SiC matrix, causing great inner stress and cracking. The observation on the similar region reveals that the carbon fibers have a gradient breaking morphology, as shown in Figure 7c. This indicates that the angle between the carbon fiber and the laser beam also influence the breaking morphology. The SEM observation of the region with carbon fibers with an axial direction parallel to the laser beam reveals the relatively ordered exfoliation of the carbon fibers, leaving a fluctuated SiC matrix surface, as shown in Figure 7d. The EDS analysis on this region reveals the relatively low oxygen content, which is almost similar to that of the original C_f_/SiC composite. Such a result implies that the oxidation during laser processing could be well controlled by the waterjet coupling.

**Figure 8:**
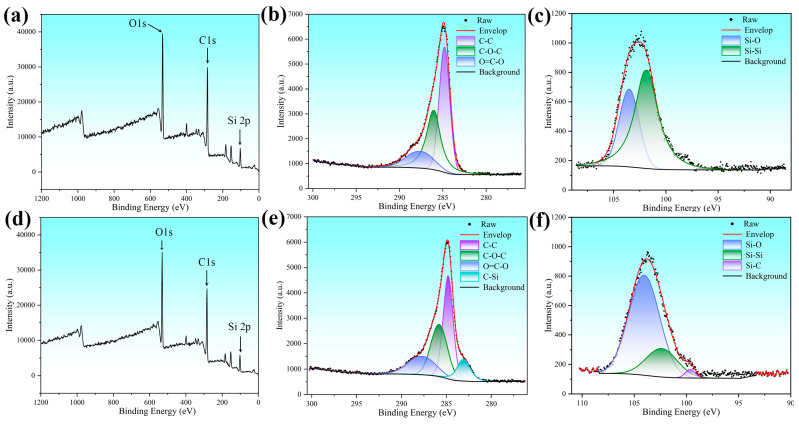
XPS analyses of the kerf surface prepared by the laser processing of the C_f_/SiC composite with the GNL and WNL modes: (**a**) Full spectrum of the XPS analysis on the kerf surface processed by GNL; (**b**) C 1s spectrum of the GNL-processed kerf surface; (**c**) Si 2p spectrum of the GNL-processed kerf surface; (**d**) Full spectrum of the XPS analysis on the kerf surface processed by WGL; (**e**) C 1s spectrum of the WGL-processed kerf surface; (**f**) Si 2p spectrum of the WGL-processed kerf surface.

To further investigate the chemical compounds formed on the kerf surface prepared by laser processing with the GNL and WNL modes, XPS analyses were applied, and the results are shown in Figure 8. Clearly, the XPS spectra of the different kerf surfaces are mainly composed of O 1s, C 1s, and Si 2p peaks, as shown in Figure 8a,d. The detailed analysis on the C 1s spectrum of the kerf surface processed by GNL reveals the presence of C-C, C-O-C, and O=C-O peaks, as shown in Figure 8b. Moreover, there are almost no C-Si peaks present. This indicates that the carbon- and oxygen-doped carbon are the main chemical bonds in the GNL-processed kerf surface. The Si 2p spectrum of the kerf surface processed by GNL mainly consists of Si-O and Si-Si peaks, as shown in Figure 8c. No Si-C peak can be observed here as well. This indicates that the silicon and its oxide should be the main phase constituents. Combined with the SEM observation, it can be deduced that the presence of oxygen promotes the oxidation in the laser-irradiated region. Due to the relatively lower melting point of the SiC compared to carbon fiber, laser irradiation firstly remelts the SiC matrix, which promotes the reaction in the melted SiC, carbon fiber, and oxygen. Therefore, the carbon tends to be oxidized and form carbon monoxide, while the SiC is transformed into SiO_2_ or silicon doped with C and O. The high content oxygen on the GNL-processed kerf surface, as observed by SEM, also confirms the deduction above. The detailed analysis of the C 1s spectrum of the kerf surface processed by WNL reveals the presence of C-C, C-O-C, O=C-O, and C-Si peaks, as shown in Figure 8e. The appearance of the C-Si peak indicates that the oxidation has been well controlled. Interestingly, the C-C, C-O-C, and O=C-O peaks of the WNL-processed kerf surface have an almost similar strength with those of the GNL-processed kerf surface. This means that these peaks can be partly attributed to the presence of oxidation in the original C_f_/SiC composite. The Si 2p spectrum of the kerf surface processed by WNL consists of Si-O, Si-Si, and Si-C peaks, as shown in Figure 8f. Compared with the Si 2p spectrum of the GNL-processed kerf surface, the strength of the Si-O peak increases, but that of the Si-Si peak decreases greatly. Besides the appearance of the Si-C peak, these results all indicate a decrease in oxidation during the WNL mode.

Combined with the SEM analyses, the reaction during the laser processing of the C_f_/SiC composite with the GNL mode can be summarized as follows. The initial laser irradiation on the C_f_/SiC composite increases the temperature of the specific region and its remelting. With the temperature increasing above 2700 °C, the SiC firstly vaporizes as reaction (1). Simultaneously, if the temperature increases further, the decomposition of the SiC occurs as reaction (2) [43]. These reactions produce the vapor of the SiC, Si, and C, which immediately react with the oxygen and form the SiO_2_ dust and CO_2_. With the heat transfer, the region adjacent to center of the laser spot is heated to a high temperature and induces the solid oxidation. Due to the differing supplying of oxygen, there are several reactions, from reactions (3)–(5). The sufficient oxygen in the reaction induces the formation of SiO_2_ and CO_2_, while the insufficient oxygen in the reaction leads to the formation of SiO and CO. If the temperature of the laser-irradiated region increases to above 3300 °C, the carbon fibers are vaporized and react with oxygen, forming CO or CO_2_. With these reactions, the surface layer of the C_f_/SiC composite tends to transform into a mixture of SiO_2_, SiO, and Si, which has a low hardness, low densification, low corrosion resistance, and low melting point. Therefore, the performance of the kerf surface processed by GNL is significantly destroyed.

(1) $$SiC\left(s\right)=SiC(g)$$

(2) $$SiC\left(s\right)=Si\left(s\right)+C(s)$$

(3) $$SiC\left(s\right)+2O_{2}\left(g\right)=SiO_{2}\left(s\right)+CO_{2}(g)$$

(4) $$SiC\left(s\right)+3/2O_{2}\left(g\right)=SiO_{2}\left(s\right)+CO(g)$$

(5) $$SiC\left(s\right)+O_{2}\left(g\right)=SiO\left(g\right)+CO\left(g\right)$$

Different from the laser processing with the GNL mode, the WNL mode makes use of the waterjet and constructs a relatively isolated laser processing environment. Even though the laser irradiation can generate air bubbles and cavitation erosion, the waterjet removes the products immediately. Therefore, a kerf surface with a high cleanliness and flatness can be achieved by laser processing with the WNL mode in the C_f_/SiC composite. Nevertheless, the isolation effect of the waterjet also results in a vacuum-like condition in the laser-irradiated micro-region at initiation stage, which promotes the occurrence of reaction (2). The presence of the Si-Si bond in the WNL-processed kerf surface also confirms such a reaction. Due to the high temperature of the laser-irradiated composite, the subsequently formed air bubbles induce some oxidation as reactions (4) and (5). The SEM analysis on the WNL-processed kerf also reveals a little increased oxygen content compared to the original C_f_/SiC composite, which should be attributed to the oxidation in the superficial layer.

### 3.3. Effect of Coupling Medium on Laser-Processed Kerf

For the laser processing of the CMC, its surface quality is mainly determined by the laser parameters and coupling medium [44,45]. The material removal rate is mainly controlled by the single-pulse energy of the laser, which, combined with the laser spot size, determine the processing efficiency. Certainly, the smaller the laser spot, the higher the processing precision, but the lower the processing efficiency. However, the overly high laser energy results in a thicker HAZ, which decreases the performance of the composite by generating inner stress. Therefore, the coupling medium plays an important role in the laser processing of the CMC. A high cooling rate and inset-protecting effect are always the basic requirements for the coupling medium, which handicaps the oxidation and restrains the HAZ.

**Figure 9:**
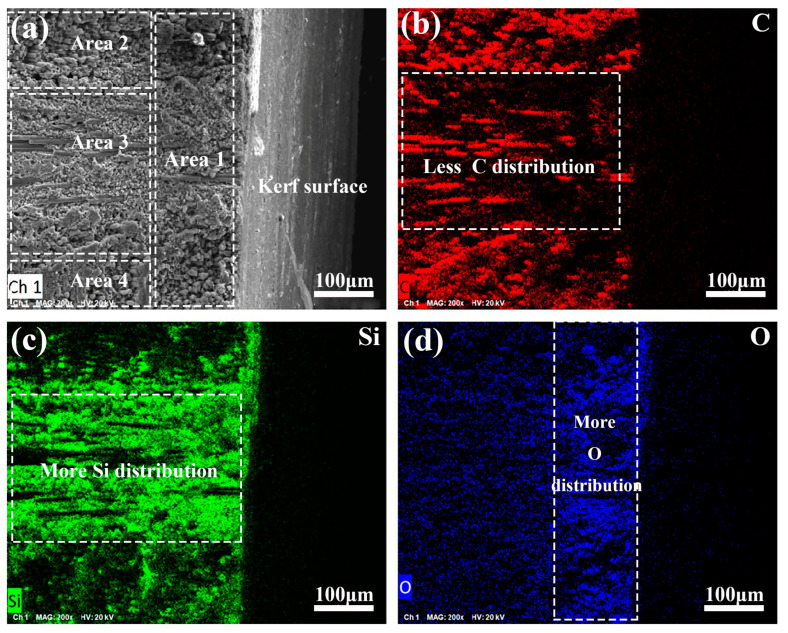
SEM analyses of the cross-section of the kerf prepared by laser processing with the GNL mode in the C_f_/SiC composite: (**a**) Typical microstructure of the cross-section; (**b**) Elemental distribution of C; (**c**) Elemental distribution of the carbon of Si; (**d**) Elemental distribution of O.

In the present research, the coupling of air and the waterjet in laser processing has exerted an obvious influence on the superficial layer of the C_f_/SiC composite, as shown in Figure 9. The cross-section of the kerf prepared by laser processing with the GNL mode can be defined in four parts, as shown in Figure 9a. Obviously, Area 1, adjacent to the kerf surface, contains a relatively coarse globular microstructure in the SiC matrix. In the inner region, the SiC matrix demonstrates a relatively fine microstructure. Based on the carbon fiber direction, the inner region can be divided into three areas. The top region, Area 2, and the bottom region, Area 4, have carbon fibers with an axial direction vertical to the cross-section, while the middle region, Area 3, has carbon fibers with an axial direction parallel to the cross-section. The elemental mapping analyses reveal that Area 1 is deficient in C but rich in Si and O, as shown in Figure 9b–d. The depth of the O-rich layer is about 200 µm. Considering the porosity of the C_f_/SiC composite, the diffusion of oxygen in the composite is closely related with the HAZ due to its promotion effect in elemental diffusion [46,47]. Due to the inner diffusion of oxygen, the silicon is preferentially reacted, forming SiO_2_. Li et al. [48] revealed that silicon oxides could be reduced by C at a temperature of 1873 K. The possible reactions of SiO_2_ and C are listed in Table 3, and the corresponding Gibbs free energy at 1873 K was calculated. It can be found that SiC, SiO, and Si are the final products. Based on these results, Si and SiO should be the main reaction products. Compared with Areas 2 and 4, the Si content in Area 3 is high, but the content of C is low. Moreover, the O content in this region is a little higher. Such a phenomenon indicates that the carbon fibers with an axial direction vertical to the kerf surface benefit from the heat transferring to the inner position, which promotes the O diffusion and reaction. On the contrary, the carbon fibers with an axial direction parallel to the kerf surface contribute little to the heat transferring to the inner position, which handicaps the oxidation and subsequent reaction.

Though the laser irradiation time is short, the high energy density and small focusing region result in ultra-high temperatures in the micro-region. The area adjacent to the kerf surface exceeds 1900 K, which induces a serial reaction with oxygen. Except for the immobilized oxygen in the composite, the oxygen for the reaction needs to be diffused from the outside, which is obviously affected by the temperature. Combined with the SEM analyses on the cross-section of the kerf prepared by the GNL mode in the C_f_/SiC composite, the anisotropic heat transfer behavior of the carbon fibers induces the different temperature distribution in the different layers. The porosity of the C_f_/SiC composite and thermal effect accelerate the O diffusion. The layer with carbon fibers with an axial direction vertical to the kerf have a higher temperature and result in a higher oxygen diffusion rate compared to the layer with carbon fibers with an axial direction parallel to the kerf. The diffused O reacts with Si, forming SiO_2_, while the subsequent reduction reaction transforms the formed SiO_2_ to Si, shown as the reaction listed in Table 3. Therefore, the regions adjacent to the kerf have a high content Si and O, especially in the layers with carbon fibers with an axial direction vertical to the kerf. As the C is oxidized into CO or CO_2_, its content in the reaction region decreases obviously. Then, it can be deduced that the region with a significantly increased O content is the main HAZ. For the kerf prepared by laser processing with the GNL mode in the C_f_/SiC composite, its HAZ is about 200 μm.

**Figure 10:**
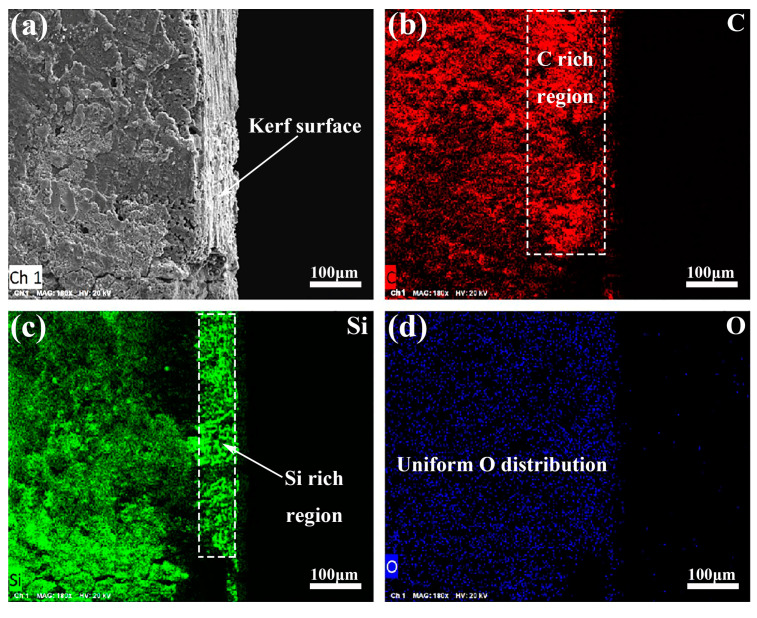
SEM analyses on the cross-section of the kerf prepared by laser processing with the WNL mode in the C_f_/SiC composite: (**a**) Typical microstructure of the cross-section; (**b**) Elemental distribution of C; (**c**) Elemental distribution of the carbon of Si; (**d**) Elemental distribution of O.

In contrast, the SEM analyses on the cross-section of the kerf prepared by laser processing with the WNL mode in the C_f_/SiC composite demonstrate different features, as shown in Figure 10. The cross-section of the kerf exhibits a similar microstructure as the original C_f_/SiC composite, indicating little influence from the laser processing, as shown in Figure 10a. The EDS analyses on the cross-section show the change in the elemental distribution, as shown in Figure 10b–d. There is an obvious enrichment of Si in the superficial layer with a depth of about 100 μm, but this region is somewhat deficient in C. Based on the analysis of the reaction in the HAZ of the laser-processed C_f_/SiC composite, this region occurs in the oxidation reaction of the SiC and reduction reaction of the formed SiO_2_. However, the O distribution in the whole observed region is relatively homogeneous. This implies that the diffused O mainly reacts and transforms into gas, releasing outwards. Moreover, there are some regions that are rich in C but deficient in Si, as shown in Figure 10b,c. Such a phenomenon should be attributed to the ultra-high-temperature and vacuum-like environment in the micro-region produced by the WNL mode [49,50]. These conditions lead to the decomposition of SiC, forming Si and C. Due to the low melting point of Si, the formed Si tends to infiltrate outside through the porous structure under the vacuum-like effect. Such a behavior also contributes to the formation of a Si-enrichment layer in the surface. In general, the presence of the waterjet in the WNL mode can well eliminate the thermal effect caused by the laser irradiation, which restrains the phase transformation and benefits the performance. Based on the thermal effect in the composite, the HAZ in the kerf prepared by WNL is less than 100 μm.

Compared with the laser-processed C_f_/SiC composite by the GNL mode, the laser processing with the WNL mode obtains a much high quality of kerf in the C_f_/SiC composite. As shown in Table 4, the advantages of the WNL-processed C_f_/SiC composite include a relatively small taper angle, low oxidation, relatively smooth surface, and small HAZ. Especially for the decrease in oxidation and HAZ, they have an important role in the processing of CMCs with a small-sized shape, because it ensures a high surface quality with fewer procedures. Such features also provide the WNL with greater potential in the processing of hard and brittle materials with insulating features. However, the C_f_/SiC composite processed by WNL still has some exposed fibers and fiber pullouts, which are detrimental to the performance. In addition, the taper feature still exists in the kerf processed by WNL, which influences the precision of the processed component. In the future, these problems need to be addressed, thereby promoting its wide application in the processing of CMCs.

**Figure 11:**
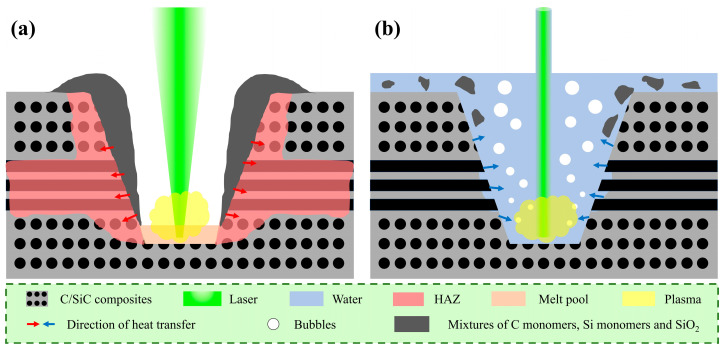
Schematic diagram illustrating the laser processing of kerfs with the GNL mode (**a**) and the WGL mode (**b**) in the C_f_/SiC composites.

Based on the analyses above, the influence of the coupling medium on the laser processing of the C_f_/SiC composite is summarized in Figure 11. During the laser processing, laser irradiation is transmitted into heat in the local region by the photothermal effect. Generally, the heat mainly concentrates in the micro-region irradiated by the laser beam, which remelts the local region and forms a plasma explosion above [51,52,53]. As in the description above, the vaporization of SiC, the decomposition of SiC, and the oxidation of SiC occurs in succession. In the kerf prepared by GNL, the spattering of the remelted SiC forms a bulge on the kerf margin and a recast layer on the kerf surface, as shown in Figure 11a. Though the helium exerts some protection on the processed composite, its effect is limited due to its small gas flow. In addition, the convection caused by the helium flow also involves more oxygen, which results in oxidation in the laser-processed region. The oxidation produces a mixture of Si oxides in the bulge and recast layers. In the kerf prepared by WNL, the waterjet isolates the air and cools down the laser-irradiated region rapidly, which restrains the oxidation effectively, as shown in Figure 11b. Even though the laser irradiation generates some air bubbles, their oxidation and cavitation erosion influence is relatively much smaller than the opening to air. In addition, the remelted composite can be taken away from the waterjet, thus producing a clean and flat surface.

Except for the kerf surface, the coupling medium also affects the microstructure of the layer under the kerf surface. Such an influence is mainly exerted by the thermally affected and inner reactions. As with the observation on the cross-section, the reactions include oxidation and reduction. As a result, the inner oxidation and subsequent reduction lead to elemental segregation, which is closely related with the size of the HAZ. Due to the anisotropic thermal transfer behavior of the carbon fiber, more heat transfers along the axial direction, which promotes the differing temperature distribution in the superficial layer of the laser-processed C_f_/SiC composite [54,55,56]. Especially in the laser processing of the C_f_/SiC composite with the GNL mode, the heat has relatively more time to dissipate and more heat is transferred to the inner position of the composite, as shown in Figure 11a. This induces a larger HAZ under the kerf, particularly in the region with carbon fibers with an axial direction vertical to the kerf surface. The long thermal dissipating time produces a larger HAZ in the kerf prepared by GNL. The coupling with the waterjet realizes the isolation effect and rapid cooling, as shown in Figure 11b. The cooling effect decreases the HAZ, but the isolation effect induces the vacuum-like condition, which promotes the decomposition of the SiC in the superficial layer. Even though the generated oxygen leads to oxidation in the superficial layer, its thickness is limited. Furthermore, such a reaction does not result in a layer with a thorough phase transformation. Therefore, the kerf prepared by the WNL mode in the C_f_/SiC composite possesses a better performance.

## 4. Conclusions

In this paper, the laser processing with the GNL and WNL modes were applied to fabricate thin kerfs in the C_f_/SiC composite. The surface morphology, microstructure, and chemical composition of the processed C_f_/SiC composite were studied comparatively. The influence of the coupling medium and corresponding mechanism were discussed. The following conclusions can be drawn:

(1) The coupling of helium in the GNL mode laser processing can make full use of the laser energy, but it results in spattering in the kerf margin and a recast layer in the kerf surface, accompanied by obvious oxidation, while the coupling of the waterjet in the WNL mode laser processing decreases the oxidation significantly and removes the remelting debris, which produces a clear and flat kerf surface.
(2) Due to the taper caused by laser energy dissipation, the single-path laser processing in the C_f_/SiC composite has a limited depth. The maximum depth of the kerf prepared by single-path laser processing with the GNL mode is about 328 μm, which can be obtained by repeating the single-path processing four times. The maximum depth of the kerf prepared by single-path laser processing with the WNL mode is about 302 μm, which can be obtained by repeating the single-path processing five times.
(3) The multi-path laser processing with the GNL and WNL modes can realize the fabrication of the through kerf in the C_f_/SiC composite, but the coupling medium obviously influences the kerf surface and microstructure of the underlying region. The kerf surface prepared by the GNL mode has a varied surface morphology, which transits from the top layer, covered with oxide particles and some cracks, to the bottom layer, featured with micro-grooves and small oxides. The kerf surface prepared by the WNL mode has a consistently smooth and clean morphology, featured with broken carbon fiber and residual SiC matrix.
(4) During the fabrication of the kerfs in the C_f_/SiC composite, the ultra-high temperature and presence of oxygen induce inner oxidation. The slow laser energy dissipation and open environment in the GNL mode results in a larger HAZ and a relatively serious oxidation, which causes local phase transformation and microstructure degradation. The isolation condition and rapid cooling in the WNL mode decreases the HAZ and restrains the oxidation, almost keeping the original microstructure. The thicknesses of the HAZ in the GNL- and WNL-processed C_f_/SiC composite are about 200 μm and 100 μm, respectively.

For the laser processing with the WNL mode, more studies should be performed to further increase the surface quality and decrease the taper formed. An improved processing precision will contribute to its application in more fields. Especially for hard and brittle materials with an insulating feature, laser processing coupled with an appropriate medium would be the best choice.

## Figures and Tables

**Table 1:** The detailed physical properties of the C_f_/SiC composite.

Diameter of Carbon Fiber (μm)	Thickness of Pyrolytic Carbon Interface Layer (nm)	Carbon Fiber Volume Fraction (%)	Density (g/cm^3^)	Porosity (%)
6~8	~300	~40	1.7	~18

**Table 2:** The detailed parameters of the WGL and GNL processing experiments.

Waterjet Velocity (m/s)	Helium Pressure (MPa)	Nozzle Diameter (μm)	Scanning Speed (mm/s)	Laser Spot Diameter (μm)	Distance Between Parallel Paths (μm)
110	0.2	100	3	30	70

**Table 3:** The reactions between C and SiO_2_ at 1873 K and the corresponding Gibbs free energy.

Reaction	$∆G_{T}^{1873}(T)$(KJ/mol)
$SiO_{2}+3C=SiC+2CO$	−9.073
$SiO_{2}+2C=Si+2CO$	−11.303
$SiO_{2}+C=SiO+CO$	−9.56
$SiO_{2}+2SiC=3Si+2CO$	−7.56

**Table 4:** Comparison of the laser processing modes of the C_f_/SiC composite by GNL and WNL.

Laser Processing Mode	GNL	WNL
Taper	Relatively high	Relatively small
Recast	Obvious and thick	Not visible
Oxidation	A great amount	Small ratio
HAZ	About 200 μm	Less than 100 μm

## Data Availability

The original contributions presented in the study are included in the article, further inquires can be directed to the corresponding authors.
